# Structural and functional annotation of the porcine immunome

**DOI:** 10.1186/1471-2164-14-332

**Published:** 2013-05-15

**Authors:** Harry D Dawson, Jane E Loveland, Géraldine Pascal, James GR Gilbert, Hirohide Uenishi, Katherine M Mann, Yongming Sang, Jie Zhang, Denise Carvalho-Silva, Toby Hunt, Matthew Hardy, Zhiliang Hu, Shu-Hong Zhao, Anna Anselmo, Hiroki Shinkai, Celine Chen, Bouabid Badaoui, Daniel Berman, Clara Amid, Mike Kay, David Lloyd, Catherine Snow, Takeya Morozumi, Ryan Pei-Yen Cheng, Megan Bystrom, Ronan Kapetanovic, John C Schwartz, Ranjit Kataria, Matthew Astley, Eric Fritz, Charles Steward, Mark Thomas, Laurens Wilming, Daisuke Toki, Alan L Archibald, Bertrand Bed’Hom, Dario Beraldi, Ting-Hua Huang, Tahar Ait-Ali, Frank Blecha, Sara Botti, Tom C Freeman, Elisabetta Giuffra, David A Hume, Joan K Lunney, Michael P Murtaugh, James M Reecy, Jennifer L Harrow, Claire Rogel-Gaillard, Christopher K Tuggle

**Affiliations:** 1 USDA-ARS, Beltsville Human Nutrition Research Center, Diet, Genomics, and Immunology Laboratory, Beltsville, MD 20705, USA; 2 Informatics Department, Wellcome Trust Sanger Institute, Wellcome Trust Genome Campus, Hinxton, Cambs CB10 1SA, UK; 3 INRA, UMR85 Physiologie de la Reproduction et des Comportements, F-37380, Nouzilly, France; 4 National Institute of Agrobiological Sciences, 2-1-2 Kannondai, Tsukuba, Ibaraki 305-8602, Japan; 5 USDA ARS BA Animal Parasitic Diseases Laboratory, Beltsville, MD 20705, USA; 6 Department of Anatomy and Physiology, College of Veterinary Medicine, Kansas State University, Manhattan, KS 66506, USA; 7 Laboratory of Animal Genetics, Breeding, and Reproduction, Huazhong Agricultural University, Wuhan 430070, PR China; 8 Department of Animal Science, Iowa State University, Ames, IA 50011, USA; 9 Parco Tecnologico Padano, Integrative Biology Unit, via A. Einstein, 26900, Lodi, Italy; 10 Institute of Japan Association for Technology in Agriculture, Forestry and Fisheries, 446-1 Ippaizuka, Kamiyokoba, Tsukuba, Ibaraki 305-0854, Japan; 11 The Roslin Institute and Royal (Dick) School of Veterinary Studies, University of Edinburgh, Easter Bush, Midlothian EH25 9RG, UK; 12 Department of Veterinary and Biomedical Sciences, University of Minnesota, 1971 Commonwealth Avenue, St. Paul, MN 55108, USA; 13 National Bureau of Animal Genetic Resources, P.B. 129, GT Road By-Pass, Karnal 132001, (Haryana), India; 14 INRA, UMR1313 Génétique Animale et Biologie Intégrative, F-78350, Jouy-en-Josas, France; 15 Current affiliation: EMBL Outstation-Hinxton, European Bioinformatics Institute, Wellcome Trust Genome Campus, Cambs CB10 1SD, UK

**Keywords:** Immune response, Porcine, Genome annotation, Co-expression network, Phylogenetic analysis, Accelerated evolution

## Abstract

**Background:**

The domestic pig is known as an excellent model for human immunology and the two species share many pathogens. Susceptibility to infectious disease is one of the major constraints on swine performance, yet the structure and function of genes comprising the pig immunome are not well-characterized. The completion of the pig genome provides the opportunity to annotate the pig immunome, and compare and contrast pig and human immune systems.

**Results:**

The Immune Response Annotation Group (IRAG) used computational curation and manual annotation of the swine genome assembly 10.2 (Sscrofa10.2) to refine the currently available automated annotation of 1,369 immunity-related genes through sequence-based comparison to genes in other species. Within these genes, we annotated 3,472 transcripts. Annotation provided evidence for gene expansions in several immune response families, and identified artiodactyl-specific expansions in the cathelicidin and type 1 Interferon families. We found gene duplications for 18 genes, including 13 immune response genes and five non-immune response genes discovered in the annotation process. Manual annotation provided evidence for many new alternative splice variants and 8 gene duplications. Over 1,100 transcripts without porcine sequence evidence were detected using cross-species annotation. We used a functional approach to discover and accurately annotate porcine immune response genes. A co-expression clustering analysis of transcriptomic data from selected experimental infections or immune stimulations of blood, macrophages or lymph nodes identified a large cluster of genes that exhibited a correlated positive response upon infection across multiple pathogens or immune stimuli. Interestingly, this gene cluster (cluster 4) is enriched for known general human immune response genes, yet contains many un-annotated porcine genes. A phylogenetic analysis of the encoded proteins of cluster 4 genes showed that 15% exhibited an accelerated evolution as compared to 4.1% across the entire genome.

**Conclusions:**

This extensive annotation dramatically extends the genome-based knowledge of the molecular genetics and structure of a major portion of the porcine immunome. Our complementary functional approach using co-expression during immune response has provided new putative immune response annotation for over 500 porcine genes. Our phylogenetic analysis of this core immunome cluster confirms rapid evolutionary change in this set of genes, and that, as in other species, such genes are important components of the pig’s adaptation to pathogen challenge over evolutionary time. These comprehensive and integrated analyses increase the value of the porcine genome sequence and provide important tools for global analyses and data-mining of the porcine immune response.

## Background

The co-evolution of host response and pathogen evasion mechanisms [1] drives variation in the response to infectious diseases at individual, population and species levels, as well as at higher order taxonomic units. Immunity-related genes are “tailored to the niche that a species occupies” and exhibit many features of positive selection, including polygeny and clustering of loci, high rates of non-synonymous substitutions (dN/dS), allele and gene conversion, generation of repertoires, rapid evolution, co-evolution, association with diseases and networking [2].

The host response to a pathogen requires concerted action of a huge set of immunity-related genes recently referred to as the immunome [3]. The immunome was first defined as the totality of rearranged antibody and antigen receptor genes present in all living individuals of a species, including variations in the somatic rearrangements [4]. That definition was further adapted to describe the whole set of genes related to both innate and acquired immunity as identified from whole genome sequencing and functional genomics studies [3]. The progress in genome sequencing of human, model and non-model animal species, including livestock species ([5-10]), now permits comparative analysis across many species [11]. Such studies have highlighted the divergence in innate immune responses between humans and mice, the most widely-studied experimental animal model [12].

The domestic pig (*Sus scrofa*), in the Suidae family within the Cetartiodactyl order of eutherian mammals, has been used a model in medical research due to its similarity to humans in size and physiology, including comparable digestive, respiratory, and immune systems (reviewed by [13,14]). The Cetartiodactyl order first appeared 60-65 million years ago, while the divergence for rodents and primates (Euarchontoglires) dates from 74 and 77 million years ago, respectively [15]. Primarily as a consequence of the fast sequence evolutionary rate of mouse, the pig has remained considerably more similar at the DNA sequence level to humans [16]. However, some immunological features are exceptional in the pig compared to humans and mice. Pigs have an inverted lymph node structure and an unusual route for lymphocyte circulation [17]. There are relatively high numbers of extra-thymic CD4/CD8 double positive T-cells [18] and resting T-cells expressing *SLA* class II molecules [19]. Pigs can have high numbers of natural killer cells [20] and γδ cells [21], harbor an unusual diversity of B-cell and antibody repertoire development [22], and have highly heritable variation in immune cell parameters [23-25].

In pigs as in many other species, the numerous *in vivo* and *in vitro* studies on host-pathogen interactions [26-34] and immunity stimulation [35,36], are now often based on functional genomics approaches such as transcriptomic approaches [37]. With such rapid accumulation of high-dimensional data on immune response, network models are becoming increasingly important in the interpretation of such experimental data [38-43]. Correlation networks based on immune response data not only permit the identification of common regulatory mechanisms through integration with promoter/flanking sequences, but also provide evidence that un-annotated genes are involved in immune response pathways [28,34,39]. Thus an important aspect of gene annotation is the integration of structural analysis of RNAs and genomes with functional data on transcriptional response to pathogens and immune stimuli.

The purpose of the Immune Response Annotation Group (IRAG) was to explore the porcine immunome by exploiting the recently available genome sequence assembly [44]. A gene list for detailed manual gene annotation using Otterlace [45,46] was compiled using Gene Ontology (GO) annotation [47] and literature sources. Analyses combined structural, evolutionary and functional approaches. We report a refined gene structure annotation on greater than 1,000 genes involved in immunity; data on positive selection pressure of a subset of the proteins predicted to be encoded by these genes; and a correlation network analysis of transcriptomic data from various disease and immunological models. These three levels of data contribute to a better characterization of the pig immunome and provide a comparative genomic appraisal across mammals.

## Results and discussion

### Extensive manual annotation of the genomic complement of porcine immune response genes

The Immune Response Annotation Group (IRAG) members used Otterlace [45,46] to manually annotate over 1,400 loci in porcine build 9 selected based on their membership in immune response processes or Gene Ontology immune response annotation. The GO term used as an inclusion criterion was “immune system process”; GO:0002376. These structural annotations were transferred over to build 10.2 and discrepancies were addressed to determine the final annotation results (Table 1). Members confirmed automated annotation of 988 known genes through manual annotation of 3,472 transcripts and 1,369 gene models; 1,554 annotated transcripts contained the full-length protein coding sequence. Twenty-six pseudogenes were also identified during the annotation.

**Table 1:** Summary of genes annotated by Immune Response Annotation Group (IRAG)

**Chromosome**	Number of genes*	**Known genes annotated**	**Number of transcripts annotated**	**Number of protein coding transcripts annotated**	**Number of complete protein coding transcripts annotated**	Number of predicted genes found to be pseudogenes**	**Number of non-organism-supported# transcripts annotated**
1	103	78	218	179	120	9	65
2	102	83	292	220	122	1	86
3	74	59	167	130	60	0	38
4	186	125	452	354	222	5	119
5	72	61	174	140	85	0	47
6	90	67	220	176	97	2	81
7	76	66	163	122	92	1	37
8	45	34	102	84	39	0	23
9	62	45	132	106	53	1	38
10	21	19	84	68	32	0	20
11	9	9	13	11	8	0	3
12	105	82	221	181	95	4	68
13	68	52	173	139	69	0	67
14	84	63	265	200	100	2	103
15	38	31	100	76	38	1	39
16	23	17	58	45	17	0	23
17	39	34	79	66	39	0	18
18	14	11	19	17	8	0	3
X	158	52	540	462	258	0	294
Total	1369	988	3472	2776	1554	26	1172

*Number of gene objects created in the Otterlace annotation system.

** Processed and non-processed pseudogenes are included.

# No porcine EST or cDNA sequence was available to create these transcript predictions.

Importantly, the cross-species alignment tools in Otterlace allowed annotation of 1,172 transcripts in the pig genome using only mRNA sequence from other species (Table 1). Such transcripts without specific porcine sequence support were often made using human cDNAs (and proteins), as there are many more human than pig sequences in the databases. The conservation between human and pig in terms of synteny is three times greater than between human and mouse [48], and the pig is more closely-related at the DNA sequence level to humans than either is to the mouse [16]. Specifically for immune gene families, recent analysis at the cDNA sequence level of pig, mouse and human has shown that the great majority of human genes that were lost through evolution in the mouse were retained in the pig. Conversely, very few mouse genes that were lost through evolution in the human were found in the pig. Comparison of expansion or contraction of orthologous gene families indicated far more similar rates and classes of genes in humans and pigs than in mice. The conservation of homology and structural motifs of 1,371 unambiguous orthologs from pigs, mice and humans revealed that the overall mean similarity to human proteins was significantly higher for pigs compared to mouse [49].

The following sections provide summaries of important groups of genes for which the manual annotation revealed new insight. One important region highly relevant to immune response, the Swine Leukocyte Antigen Complex (MHC), has been previously annotated in detail [50] and will not be discussed here.

#### T cell receptor (TCR)

Genes in the TCR complex possess highly repetitive sequences, so that it is difficult to generate correctly reconstructed loci from shotgun sequencing with low redundancy or short-read next-generation sequencing. Therefore, intensive sequencing efforts were applied to the *TRA/TRD* (T cell receptor α/δ) and *TRB* (T cell receptor β) loci. The pig *TRD* locus is embedded in *TRA*, and D (diversity) (Dδ) and J (joining) segments (Jδ), and genes encoding the C (constant) region of TCR δ (Cδ) are located between the V (variable) segments of TCR α/δ (Vα/Vδ) and J genes of TCR α (Jα), as observed in other mammals. All of the human 61 Jα segments correspond to those of pig, and most of mouse Jα can be allocated to orthologs in pig. These indicate functional similarity of the TCR α molecule between human, mouse and pig [51]. On the other hand, the pig TCR δ gene (*TRD*) has a more complicated structure than those of human and mouse. Pig has at least 6 Dδ genes, while human and mouse have 3 and 2, respectively. The pig Dδ genes are frequently used in functional TCR δ transcripts with up to 4 concatenated domains [52]. Thus, the pig can generate a high diversity of TCR δ chain molecules to cope with antigens, which may be related to the fact that the percentage of γδ T cells in peripheral blood is much higher in pig than in human and mouse [17]. As for *TRB*, pig has 3 functional Dβ-Jβ-Cβ units, while human and mouse each have 2 units [53].

#### Immunoglobulin (IGH and IGL)

*IGHV* gene diversity is highly restricted, as in cattle, but all known porcine *IGHV* genes belong to a single family, *IGHV3*, whereas all cattle *IGHV* belong to *IGHV4*[54,55]. The lambda light chain (IGL) locus on SSC14 contains 22 *IGLV* gene segments, with 9 appearing functional. The locus is organized into two distinct clusters, a constant (C)-proximal cluster containing *IGLV3* family members, and a C-distal cluster containing *IGLV8* and *IGLV5* family members [56]. The porcine *IGLV8* subgroup genes have recently expanded, suggesting a particularly effective role in immunity to porcine-specific pathogens, especially since *IGLV* expression is nearly exclusively restricted to the *IGLV3* and *IGLV8*[56,57]. The C-distal IGLV cluster also contains three non-functional *IGLV1* family members that are orthologous to *IGLV* that are exclusively expressed in cattle [58]. The *IGL* locus contains three tandem *IGLJ–IGLC* cassettes, two of which are functional, and a fourth *IGLJ* with no corresponding *IGLC*.

The kappa light chain (*IGK*) locus on SSC3 is comprised of at least 14 *IGKV* genes, of which 9 are functional and belong to either the *IGKV1* or *IGKV2* gene families, five *IGKJ* genes that lie 27.9 kb downstream, and a single *IGKC* gene [59]. Polymorphisms within the individual Duroc sow that was genome sequenced revealed alleles that differed by as much as 16 percent among *IGKV* genes and as much as eight percent in amino acid sequence among *IGLV* genes.

The porcine immunoglobulin genes have evolved such that specific gene families have expanded and contracted with respect to other species, notably cattle. The high level of allelic variation found within the antibody light chain loci substantially expands the population diversity of the porcine antibody repertoire [56,59]. In the kappa locus, in particular, many *IGKV2* family members share specific parts of coding regions, such as complementary determining region 1, between genes but not between alleles. Thus, germline gene conversion may provide a mechanistic basis for the high level of *IGKV* allelic variation.

#### Killer immunoglobulin-like receptor (KIR)

Pigs appear to have a single *KIR* gene in contrast to cattle, horses and primates in which there is an expanded *KIR* gene family [60]. In rodents, the functionally equivalent receptors are encoded by the expanded gene family of killer cell lectin-like receptor (*KLR*) genes of which *Klra1* (*Ly49*) has 11 paralogues whilst pigs have a single *LY49* gene (*KLRA1*) with two putative orthologs. The limited NK cell repertoire in pigs is not linked with any deficiency in NK cell numbers [23] but there is evidence for a connection between high variability between individual animals and performance under low health status conditions [61,62]. One would anticipate, given the function of NK cell receptors in recognition of MHC class 1 proteins, that there must be some unique feature to this interaction in the pig to allow NK cells to function irrespective of unlinked polymorphism and the *SLA* loci.

### Immune gene family expansion

A preliminary analysis of immune gene families has previously compared humans, mice and pigs [49]. In the current analysis, artiodactyl-associated families were also included based upon expansions noted in the bovine genome [9]. Our porcine genome analyses show that some of these expansions are also present in the porcine genome, indicating an artiodactyl-specific expansion. Other expansions are not present in the pig genome, providing additional support for a ruminant-specific expansion [9]. Results are summarized in Table 2 and full details shown in Additional file 1: Table S1.

**Table 2:** Greater pig-human similarity revealed by gene family analysis

**Family description (SF: superfamily)**	Number of genes found for each family per species*			
	**Human**	**Mouse**	**Cow**	**Pig**
ADP-ribosyltransferase/VIP2 SF	4	5 (1)	9	4
Beta Defensin SF	39 (9)	51 (1)	~106 (7)	34 (2)
BPI SF	12 (2)	16	18	14 (2)
C-type Lysozyme/LYZ1 SF	9	9	16	7
Cathelicidin SF	1	1	10	10
CCL Chemokine	28 (1)	39 (5)	22	21
CD1 SF	5	2	15 (2)	4 (1)
CD163/WC1 SF	3	4	15	4
CLECT SF (inclusive)	85 (3)	126 (6)	89	76
CLECT SF, AGP and DCR Subfamily	16	24 (1)	14	13
CLECT SF, Collectin Subfamily	7 (2)	7	10	7
CLECT SF, NK Cell Receptor Subfamily	24 (1)	57 (5)	31	23
CLECT SF, Reg Subfamily	5	7	3	3
Cytidine Deaminase-like SF	11 (0)	5 (0)	6 (0)	5 (0)
GH18 Chitinase Like SF	6 (1)	9 (1)	8	7
Granzyme/ MC Tryptase/SP SF	17 (1)	26 (0)	22 (0)	18 (0)
Immunity Related GTP-ase SF	3 (2)	19 (4)	4 (0)	4 (0)
NLR and Pyrin SF	31 (4)	43 (8)	23	25
Resistin SF	2	4	1	2
RNase A Family	14	22 (6)	16 (1)	13 (1)
S100 SF	21	17 (1)	19	20
SAA SF	4 (1)	5	6	6
SLAM SF	9 (1)	9 (0)	12 (0)	11 (0)
Toll Like Receptor	10 (3)	12 (1)	10	10 (2)
TRIM E3 Up Ligase SF, TRIM5 Subfamily	4	10 (1)	5	3
Type I Interferon (inclusive)	17 (12)	25 (2)	51 (13)	39 (16)
Type I Interferon, Alpha Subfamily	13 (4)	13 (1)	19 (3)	18 (9)
Type I Interferon, Beta Subfamily	1	1	8 (1)	1
Type I Interferon, Delta Subfamily	0	0	0	11 (2)
Type I Interferon, Epsilon Subfamily	1	1	0	1
Type I Interferon, Kappa Subfamily	1	1	1	1
Type I Interferon, Omega Subfamily	1 (8)	0	19 (7)	7 (5)
Type I Interferon, Tau Subfamily	0	0	4 (2)	0
Type I Interferon, Zeta Subfamily	0	9 (1)	0	0
ULBP SF	6	2	12	7

* Numbers of confirmed pseudogenes are shown in parentheses. AGP and DCR: Asialo- glycoprotein and DC Receptor; MC: Mast Cell; SP: Serine Protease; Up: Ubiquitin-protein.

The cathelicidin gene family was expanded, with 10 genes compared to only one in human and mouse. The expansion appears to be artiodactyl-specific since cattle also have 10 genes [9].

Thirty-four beta defensin genes were detected in the swine genome assembly, similar to the human genome (39 genes), but substantially less than the >100 beta-defensin genes reported from cattle. A recent report annotated 29 porcine defensins in the high-throughput genome sequences (HTGS) pre-assembly (Choi, 2012), which indicates that our annotation of 34 for the current genome assembly adds to the previous annotation of this family. For this work, we tested the procedure based on hidden-Markov model (HMM) that was used in the bovine genome project [9], but primarily used our curation protocol established for genome-wide annotation of beta-defensin families in the human, chimpanzee, mouse, rat, and dog [63]. As a more sensitive procedure, HMM could overestimate the functional gene numbers without manual curation. Thus far, we suggest that the composition of the porcine beta-defensin family is more similar to human (39 genes) than bovine (106 genes). A similar result is observed for the C-type lysozyme family in pigs, which has 7 genes, while 16, 9 and 9 are found in the bovine, human and mouse genomes, respectively. Thus, our analysis of the second artiodactyl genome indicates that beta-defensin and C-type lysozyme family expansions observed in cattle may be ruminant-specific adaptations [9]. Because pigs are omnivores and cows are herbivores, it is tempting to speculate that these differences may be due to different exposure to gut microbiota.

Pigs have 39 type I interferon (*IFN*) genes, twice the number in human, as well as 16 pseudogenes. Cattle have 51 type I *IFN* genes (13 pseudogenes). This expansion is focused on interferon subtypes *IFNW* and *IFND*; pigs (p) and cattle (c) share novel subtypes of *IFND* (p), *IFNT *(c), *IFNAW* (p & c) and many more isoforms of *IFNW*. Thus, expansion of interferon genes is not ruminant-specific as previously proposed [9], although duplication within some specific sub-families appears to be either bovine- or pig-specific.

Data presented in Table 2 represents an expanded analysis of the gene families that were presented in the recent porcine genome paper [44]. Four additional gene families, *SLAM* Superfamily, Granzyme/Mast Cell Tryptase/Serine Protease Superfamily, Cytidine Deaminase-like Superfamily and Immunity Related Guanosine Triphosphatase Superfamily are included. These new analyses reveal a slight expansion in the *SLAM* superfamily in cow and pig relative to human, a relatively large expansion in the Granzyme/Mast Cell Tryptase/Serine Protease Superfamily in mouse and cow relative to humans and pigs, and an extremely large expansion in the Immunity Related Guanosine Triphosphatase Superfamily in mouse relative to the other 3 species. In contrast, the number of Cytidine Deaminase-like Superfamily members is human is twice that found in pigs, mice and cows.

The total number of pig, mouse and cow gene families that have undergone expansion of >25% of family members relative to human are 8, 17 and 14, respectively (Table 2, Additional file 1: Table S1). Thus familial gene expansion in pigs relative to humans has occurred at half the rate of mice and cows. Conversely, the total number of gene families in pig, mouse and cow that have undergone contraction of >25% of family members relative to human are 6, 4 and 4, respectively. Familial gene contraction in pigs relative to humans has occurred at roughly the same rate of mice and cows.

**Figure 1:**
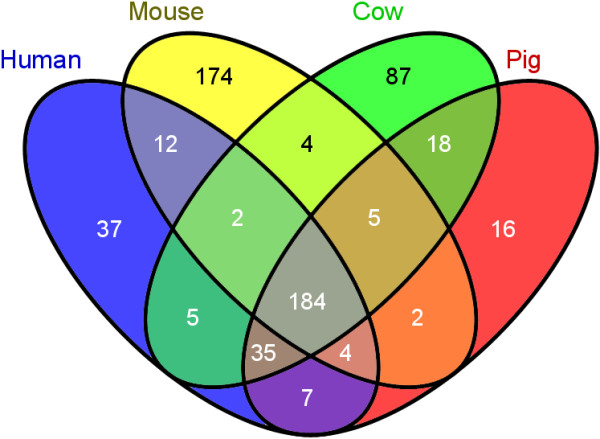
**Greater pig-human similarity revealed by orthology preservation analysis.** As shown in the graph, pigs have 11-, 6- and 2- fold less unique genes than do the mouse, cow or human.

An additional analysis of orthology preservation of familial gene expansion for the four species deriving from the family member expansion analysis revealed that 1:1 orthology conservation was found for 184 of the 597 genes (31%) (Figure 1). Mice had the largest number of unique genes (174), more than twice the number found in cattle (87), and more than all of the others combined. In contrast, pigs have 11-, 5- and 2-fold fewer unique genes compared to the mouse, cow and human, respectively. Pair-wise analysis indicates that pigs and cows share 18 genes that are not found in humans or mice (Figure 1). These genes are members of the BPI Superfamily, BPIFB5 and BPIFB9; Cathelicidin Superfamily CATHL1 (PR39), CATHL2 (NPG1), CATHL4 (NPG3), CATHL5 (NPG4), CATHL6 (NPG5), CATHL7 (PF1); CCL Chemokine Superfamily, CCL3L2; CD163/WC1 Superfamily, LOC100337197; CLECT Superfamily, KLRJ1 and PRG3L1; Granzyme/Mast Cell Tryptase/Serine Protease Superfamily, GZMAL (LOC100233183), MCPT3; Immunity Related Guanosine Triphosphatase Superfamily, IRGCL1; NLR and Pyrin Superfamily, NLRP12L; and RNase A Family, BRB and LOC782739 (RNASE15, RNASE4L).

Furthermore, analysis of three species at a time indicates that humans and pigs share 42 genes that are not found in mice; mice and humans share 14 genes that are not found in pigs; and mice and pigs share 7 genes that are not found in humans (Figure 1). These conclusions must be tempered with the observation that the porcine genome is still incomplete and additional family members may be discovered. Indeed, in the course of looking for known genes in the current porcine genome build, we identified genes that do not appear in the build 10.2 assembly; please see Additional file 2: Table S2. However, these similarities, especially for pig and human, further reinforce the use of the pig as a large animal model of the immune response of humans.

### Gene duplication

In the course of annotating the immune response gene families shown in Table 2, we found indication of gene duplication and pseudogenes in the build 10.2 assembly. A summary of putative true gene duplications is shown in Additional file 3: Table S3. Using extreme sequence similarity (approximately 99% identity) as a metric, many duplications (298) appear to be due to assembly artifacts (Additional file 4: Table S4). These artifactually duplicated genes fall into 3 different categories: on the same chromosome and mapped to the same assembly scaffold, on the same chromosome and mapped to a different assembly scaffold, and on different chromosomes. The 18 duplications that appear on different chromosomes are especially problematic because of the need to assure BAC specificity. One gene, *TNFRSF10A*, which is listed provisionally here as artifactually duplicated, proved especially problematic since there is equal evidence for both artifactual and true duplication; directed studies will be necessary to determine the nature of this duplication.

Evidence for the true duplication of 13 immune-related genes: *ATF4, CD36, CD68, CD163, CRP, DDX3X, GSTP1, GZMA, IFIT1, IL1B, IRGC, ITLN2*, and *OAS1*, and 5 non-immune genes, appears unequivocal (Additional file 3: Table S3). Interestingly, the *IL1B* gene duplication, in which evidence for a partial duplication had been reported [64], is unique in mammals. Predicted proteins expressed from the *IL1B* gene and its duplicate (*IL1BL*) are both 267 aa in length, but only 86% identical. Further, their mRNAs have different expression patterns in adult and embryonic tissues, and different responses to endotoxin in macrophages (Gong, Tuggle et al., manuscript in preparation). Unigene expression profiling of other true duplicated genes indicates that they are differentially expressed. For example the macrophage scavenger receptor *CD68* is expressed primarily in adipose tissue, blood, lung, mammary gland, and ovary, whereas the porcine-restricted duplicated gene with an unknown function, *CD68L*, has a wider tissue distribution with expression in adipose tissue, adrenal gland, blood, cartilage, heart, intestine, lung, lymph node, muscle, ovary, placenta, skin, spleen, thymus and trachea. The pattern recognition receptor *CD36* is highly expressed primarily in adipose tissue, heart, mammary gland and muscle. The truncated protein *CD36L,* with an unknown function, is also duplicated in the cow and is expressed at lower levels in blood, bone marrow liver, lung and mammary gland. The differential expression patterns of these genes support their phylogenetic and functional divergence.

### Functional annotation of immune genes: finding genes with immune response patterns similar to known immune system genes

**Figure 2:**
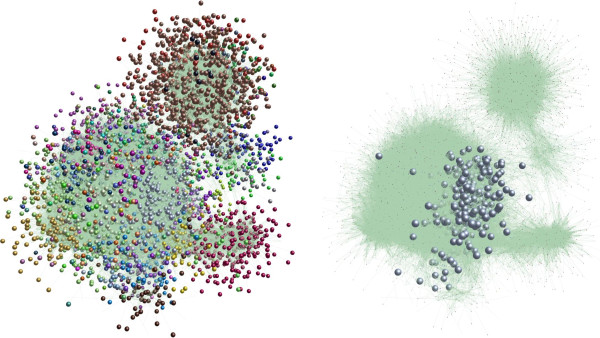
**Biolayout Express**^**3D **^**co-expression network of immune response expression patterns.** On the left is shown the network. Nodes assigned to each MCL cluster are shown in a different color. MCL is a Markov Clustering (MCL) algorithm for graph clustering (micans.org/mcl); see also http://www.biolayout.org. At right, the immune-response gene enriched cluster 4 is highlighted by increasing the size of nodes for Cluster 4 and reduction of node size in all other clusters.

Co-expression of genes can be used to provide evidence for membership in specific processes, such as immune response, when a substantial proportion of the members of an expression cluster have similar functions. We collected all data reported for the 24K Affymetrix Genechip for experiments with an immune component such as infection of tissues or cells with bacteria, viruses, or stimulation with lipopolysaccharide. Using this targeted set of 188 chips (Additional file 5: Table S5), which included public data as well as several un-published datasets from our groups, we calculated a within-group correlation for each experimental dataset. This approach emphasizes the response to pathogen/stimulus (see Methods). These correlations were then used in the co-expression network tool BioLayout *Express*^3D^ ([38,65]) to generate and visualize a transcriptome network for porcine generic immune response (Figure 2).

**Figure 3:**
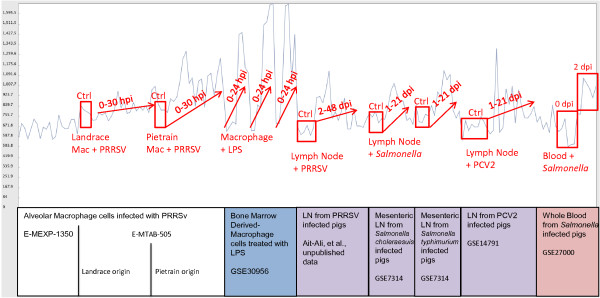
**Expression pattern of MCL cluster 4 shows gene activation after immune stimulation/infection in multiple experimental datasets.** In light blue is shown the average expression of the 619 probesets in cluster 4 from Figure 2. Details on each dataset are shown below the graph; some example patterns are highlighted in red; specific dataset patterns are boxed to show contrasts of interest. LN: lymph node; PRRSV: porcine respiratory and reproductive syndrome virus; PCV2: porcine circovirus type 2; Mac: alveolar macrophage; Ctrl: Control (uninfected) sample. Hpi: hours post infection or inoculation; Dpi: days post infection or inoculation. LPS: lipopolysaccharide.

Using this correlation network, we then identified clusters with GO term annotation enrichment related to immune response. Significance of enrichment was determined as described in Methods. In Additional file 5: Table S5 (all possible GO term annotations) and Additional file 6: Table S6 (specific annotations, see below), we document these results. Cluster 4, with 619 probesets representing at least 511 transcripts, was significantly enriched for many GO terms relevant to immune response pathways, including type I interferon-mediated signaling pathway and cytokine-mediated signaling pathway, as well as response to virus, and proteasome core complex. Cluster 4 is highlighted in Figure 2, and includes many immune-related genes such as *IL15, JAK2, IRF2, IRF7, IRF9, IFIT 1, IFIT2, IFIT3, CD40, CD47, CD86*, many *STAT*, *PSMB* and *CASP* gene family members, *MX1, MX2, CXCL16, CCRL2, WARS, SLC11A1,* and complement genes *C1R, C1S*, and *C2*. Of these 619 probesets, 96 are annotated with the GO term originally used as a major criterion for the IRAG gene list (GO: 0002376; immune system process), which is a 2.3-fold enrichment (Fisher’s exact two-tailed P value < 0.0001). This cluster is also three-fold enriched (Fisher’s exact two-tailed P value < 0.0001) for porcine orthologs of common immune response genes identified by Jenner and Young based on a meta-analysis of microarray data from a number of pathogens/ immune stimulations of several human cell types [66]. Thus cluster 4 clearly is enriched for a large number of genes likely to be involved in the immune response of swine. Importantly, most of these probesets are not already annotated as immune response genes, as only 16% have the GO: 0002376 term annotation. Further, the average RNA level for these 619 probesets across the immune response datasets is shown in Figure 3. The pattern is clearly one of activation upon infection or treatment, as global increases in expression levels are seen in many datasets upon immune stimulation. Thus this correlation analysis provides evidence for the involvement of many new genes in the porcine immune response.

A second cluster with enrichment of GO:0002376 is cluster 17 (p < 0.0001). As with cluster 4, cluster 17 is also enriched for Jenner-Young (J-Y) IR genes as well as genes manually annotated as “innate immune” genes in InnateDB (http://www.innatedb.com; Additional file 7: Table S7). For the global GO annotation, only anti-apoptosis was found significantly enriched (annotated for the genes *BIRC3, FAS, MCL1, NFKBIA, TNF, PIM2*). Genes in this smaller cluster of 81 members include several negative regulators of innate/inflammatory pathways (*ATF3, IL1RN, NFKBIA, NFKBIZ, SOCS1, SOCS3*) (Additional file 6: Table S6). The pattern of expression of these genes is similar to that of cluster 4, with cluster 17 genes clearly activated in LPS-stimulated macrophages and in lymph nodes or blood from pigs infected with *Salmonella* (Additional file 8: Figure S1). Interestingly, in contrast to cluster 4, several datasets did not show activation of genes represented by the probesets in cluster 17 genes on average, including lymph nodes infected with PCV2 or PRRSV. A third cluster contains 48 probesets (cluster 26), containing probesets representing several T-cell related genes (*CD2, CD3D, CD3E, CD8A, CD8B, LCK*) as well as other immunity genes such as *IL7R, GZMB* and *STAT4*. This cluster appears to document gene expression specific to T cells and neutrophils, as expression is detected in lymph node and whole blood datasets, and absent in macrophages (Additional file 9: Figure S2). The global annotation identified regulation of immune response as well as several T cell receptor terms as enriched in this cluster (Additional file 6: Table S6). The pattern of expression is not clearly correlated with response to immune stimulation, which is consistent with the observation that these genes are not enriched for J-Y IR or InnateDB genes, but are enriched for genes with GO:0002376 annotation (Additional file 7: Table S7). Finally, cluster 48 is enriched only for probesets annotated with the GO:0002376 term, and shows early and robust induction in macrophages infected with PRRSV and in lymph nodes in animals infected with *Salmonella enterica* serovar Typhimurium, but variable low to modest induction in macrophages by LPS (Additional file 10: Figure S3) and other challenges. This cluster of 24 probesets includes a number representing MHC genes (*DRA, DRB* and *DMA* families) as well as *CFP, CYBA, Ly86* and *IRF8*. Comprehensive GO annotation shows this cluster is enriched for interferon gamma-mediated signaling pathway, T cell receptor signaling pathway, cytokine-mediated signaling pathway, and immune response genes (Additional file 6: Table S6).

The established gene lists for generic immune responses at the level of the transcriptome can be used to improve the annotation of a large number of genes/transcripts in the porcine genome related to immune response. Especially for cluster 4, which is significantly enriched for probesets annotated as human immune response genes, these data provide foundational information that can be used for human-pig comparisons at several levels. For example, global comparisons of promoter sequence elements between pig and other species can be performed using draft genome assembly information, as shown recently for an analysis of the *CYP27B1* gene promoter [36]. Previously, substitution of human orthologous promoters was useful in prediction of sets of NFκB target genes in the pig [28]. Given the recent expansion of transcriptomic datasets on immune response, especially for those analyzing response to infection of specific tissues or cell types, the pig genome will now be invaluable in bioinformatic approaches to recognize known and novel regulatory motifs in immune response genes. Prior annotation as immune response genes, as demonstrated herein, will provide further confidence for genes clustered by their transcriptomic response to an immune stimulation.

### An accelerated rate of evolution for immunity-related genes

As recently reported [44], an analysis of predicted rates of evolutionary change was carried out on a randomly selected subset of 158 immunity-related pig proteins from the IRAG annotated gene set. This analysis showed rates of positive selection between 12.7 and 17.1%, depending on the analysis method (PRANK or MUSCLE, see Methods). To confirm and extend this significant increase in the rate of positive selection for immunity-related genes in swine, we analyzed the proteins present in the cluster 4, found in the above analysis to be significantly enriched for probesets annotated as immunity-related genes. A set of 251 proteins was analyzed with the PhyleasProg web server [67], and a subset of 242 proteins having at least 10 orthologs and being compatible with threshold of statistical significance was included in final analyses.

Among these 242 proteins, 37 proteins are under positive selection with q <0.05, and 42 with q <0.10; i.e. 15% and 17% respectively. Thus, Cluster 4 is as rich in positively selected proteins, as was the subset of 158 immunity-related pig proteins recently published in the swine genome sequence paper [44]. At the whole genome level, it has been reported that the rate of positive selection, computed on different types of data and different methods, is 1.1% in human, 1.7% in chimpanzee [68], close to 5% in cattle, dog and horse (David Enard, personal communication), and 4.1% in pig (348 genes under positive selection out of 8,418 1:1 orthologs between human, mouse, dog, horse, cow and pig) [44]. Our results show a significant increase in the rate of positive selection for immunity-related genes in swine.

These results confirm that positively selected genes in swine are enriched for roles in defense and immunity in mammals, as already shown in human [1], cow [9], five other mammals [69], as well as birds like the Zebra finch [70]. Other functions are also reported as privileged targets for an accelerated evolutionary rate of related genes in mammals, such as reproduction, taste perception, chemosensory reception [69], and olfaction, as recently shown in pig [44].

**Figure 4:**
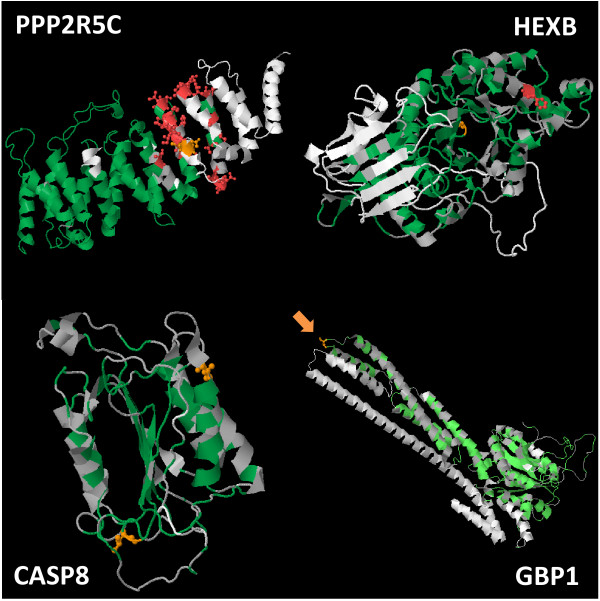
**Results of positive selection calculation are visualized onto 3D structure of PPP2R5C, HEXB, GBP1 and CASP8 pig proteins.** The color scale from light to dark green represents purifying selection, while orange and red represent positive selection with posterior probabilities greater than 95% or 99%, respectively. The orange arrow points to the serine at position 427 of GBP1 protein that is under positive selection.

By branch-site analysis, we detected an accelerated evolution of several amino acids specific to pig (positive selection on pig branch only) in 17 proteins (7% of subset of proteins, cluster 4), including SPPL2A, JAK2, PPP2R5C, CHD-1, TSPAN13, NMT1, GBP1, HEXB, FAM26F, LMAN2L, ANKMY2, PHF20L1, DDX60, PDE8A, LCP2, USP25, SLC24A6 (Additional file 11: Table S8). The projections of amino acids under positive selection onto 3D structure of the four proteins CASP8, HEXB, GBP1 and PPP2R5C are shown via PhyleasProg web server (Figure 4). The PPP25RC protein is known as the protein phosphatase 2, regulatory subunit B’, gamma and is 496 amino acids long (Ensembl ID ENSSSCP00000002749). Within a segment of 50 amino acids from position 255 to 312, a total of 25 amino acids were found under positive selection. Conversely, amino acids were found under purifying selection from position 1 to 254. (Additional file 12: Figure S4A). The InterProScan revealed matches with the protein phosphatase 2A, regulatory B subunit, B56 (from position 1 to 453, 1 to 476 or 1 to 410, by PIR, PANTHER or PFAM methods, respectively), and an Armadillo-type fold from position 6 to 397 by the superfamily method. These striking results suggest two potentially distinct subdomains for the protein PPP25RC in swine. The HEXB protein, hexosaminidase B (beta polypeptide), is 538 amino acids long in swine (Ensembl ID ENSSSCP00000014965). Hexosaminidase B is the beta subunit of the lysosomal enzyme beta-hexosaminidase that, together with the cofactor GM2 activator protein, catalyzes the degradation of the ganglioside GM2, and other molecules containing terminal N-acetylhexosamines. Two amino acids were found under positive selection at positions 191 and 370. Both amino acids map to the glycoside hydrolase catalytic domain (Additional file 12: Figure S4B). At position 191, the amino acid maps also to a beta-hexosaminidase subunit related to beta-N-acetylhexosaminidase activity (GO:0004563). The CASP8 protein, known as the caspase 8, apoptosis-related cysteine peptidase comprises 252 amino acids in swine (Ensembl ID ENSSSCP00000026484) and has also been found under accelerated evolution rate in human (see Additional file 11: Table S8, [1]). Two amino acids were found under positive selection at positions 122 and 226. The amino acid at position 122 specifically maps to a predicted domain referred to as Domain Peptidase C14, caspase precursor p45, core-IL1BCENZYME (Additional file 12: Figure S4C). The GBP1 protein is known as the guanylate binding protein 1, interferon-inducible and is 590 amino acids long (Ensembl ID ENSSSCP00000007381). A unique amino acid has been found under positive selection at position 427, in the guanylate binding protein, C-terminal domain (Additional file 12: Figure S4D).

The availability of whole genome sequences has paved the way for renewed approaches to study the molecular signatures of natural selection at unprecedented scales [71]. In addition, amino acids found under positive selection are highly interesting candidates to target for further biological analyses and understanding of the link between structures and functions. In genetic studies aimed at identifying nucleotide polymorphisms involved in the variation of target traits, the analysis of evolutionary constraints at candidate mutations should provide a fundamental, additional layer of information.

## Conclusions

Our computationally-facilitated, manual annotation of immune response genes provided expert-level curation of 1,369 gene models and 3,472 transcripts, of which 1,172 annotated using sequence available only from other organisms. This extensive annotation provided evidence for gene expansions in several immune response families, and identified artiodactyl-specific expansions in the cathelicidin and type 1 Interferon families. We found gene duplications for 18 genes, including 13 immune response genes and five non-immune response genes discovered in the annotation process. We used a functional approach to discover and accurately annotate porcine immune response genes. Using co-expression analysis of transcriptional profiling data from studies on blood, macrophages, as well as lymph nodes, we identified a large cluster (n = 619 probesets) that exhibited a correlated positive response upon infection across a number of pathogens or due to different immune stimuli. Interestingly, this gene cluster (Cluster 4) is enriched for known general human immune response genes [66], yet only 16% of these genes have been annotated as immune response in the Gene Ontology project. Overall this approach has provided new putative immune response annotation for over 500 porcine genes. A phylogenetic analysis of the encoded proteins of Cluster 4 genes showed high rates of evolutionary change at the amino acid level, confirming the hypothesis that such genes are important components of the pig’s adaptation to pathogen challenge over evolutionary time. These comprehensive and integrated analyses increase the value of the porcine genome sequence and provide important tools for global analyses and data-mining of the porcine immune response.

## Methods

### Selection of genes and corresponding BACs to annotate

To enable identification of BAC sequences for annotation, we constructed a priority pig gene list to facilitate the connection of gene name to genome/BAC locations. These genes were selected to represent as many genes in the immunome as possible, based primarily on immune response GO annotation (“immune system process”; GO:0002376), but also included genes identified as response to immune stimulations or infections in human cells [66] or fast-evolving human immune genes [1]. Briefly, BAC names were extracted from ftp download pig.embl files and matched with their clone names. Their coordinate locations on the pig assembly were obtained through an interactive query from the Sanger web portal. Mapping information obtained included finished BAC clones, in-process FPC clones and un-finished clones. Among 4,245 initially proposed immune genes to annotate, only 2,990 were found on the Ensembl predicted gene list. The remaining genes were mapped using cDNA sequences to the pig assembly. With several updates from various sources, the final list contained 4,347 immune genes. A GBrowse portal displayed the alignments of the genes and BAC clones. With a coordinate-based query mechanism, an interactive web portal (http://www.animalgenome.org/cgi-bin/host/ssc/gene2bacs) was set up on a MySQL database-enabled system for curators to locate the clones of interest. The portal also provided ortholog genes and Refseq match information to assist annotation.

### Annotation methods using new Otterlace software

The Otterlace/Zmap [45,46] manual annotation software was used by authorized external users via the Wellcome Trust Sanger Institute (WTSI) SingleSignOn system using their Institute email addresses. The manual annotation approach used was that of the “Gatekeeper” [45] where the external annotation is subjected to integrated QC within the software followed by extensive QC from professional annotation staff in the human and vertebrate analysis and annotation (HAVANA) team.

When a clone of interest was identified, it was linked to the assembly sequence chooser in Otterlace. This region of the chromosome was opened by the annotator and large-scale data analysis from our annotation pipeline [45], such as searching mRNA, EST and Protein sequence libraries, was performed on WTSI systems. These analyses, together with Ensembl predictions were then used to aid the annotation. The annotated gene objects were classified into a condensed version of biotypes developed within the HAVANA team, and detailed in full in our annotation guidelines (http://www.sanger.ac.uk/research/projects/vertebrategenome/havana). This is due to the relative scarcity of pig mRNA and SwissProt entries that are required to make a coding locus biotype Known_CDS, so many more Novel_CDSs were made from cross-species mRNA evidence. The HUGO Gene Nomenclature Committee (HGNC, http://www.genenames.org/) [72] naming convention was used whenever possible for those pig genes. Where there were potential duplications, the HAVANA naming conventions were followed (see guidelines). Annotation results are summarized by chromosome in Table 1.

### Gene family selection and description of ortholog checks

Across four mammalian genomes (human, mouse, bovine and porcine), we identified a number of expansions in gene families important in the immune system. We targeted several gene families for a detailed analysis of expansions across species; families were chosen from a preliminary analysis done in humans, mice and pigs [49]. Artiodactyl-associated families were included based upon expansions noted in the bovine genome [9]. Unambiguous 1:1 orthologs for each species were initially determined from the corresponding human or mouse gene in Ensembl. For each gene, additional family members were determined by including genes that were listed as ambiguous orthologs (1:many) or by a separate Ensembl within species search for paralogs. Each Ensembl predicted gene transcript was aligned against the NCBI reference sequence database using BLAST [73] to determine the corresponding NCBI loci and reference sequence or other family members that may have been missed due to areas of the genome that were not sequenced. Results are summarized in Table 2 and detailed in Additional file 1: Table S1. These data were used to extract a gene list of 597 unambiguous orthologs across the four species that were used for Venn analysis using Venny (http://bioinfogp.cnb.csic.es/tools/venny/index.html) (Figure 1). Defensins or interferons were not included in this analysis because 1:1 orthologies could not be assigned for the great majority of genes, due to the extremely high similarity of these gene family members within and across species. Genes for which there is evidence for transcription but that are missing from genome build 10.2 are summarized in Additional file 2: Table S2.

### Duplication analysis

Genes that yielded a one:many relationship during the orthology search were subjected to an additional round of BLAST analysis. Artifactual duplication status was designated when genes possessed approximately 99% identity at the nucleotide level and were in a cluster of proximal genes tandemly duplicated at the same level of identity. Artifactual duplications are reported in Additional file 4: Table S4.

### Co-expression analysis of immune response gene expression profiling datasets

Gene expression profiling datasets for the Affymetrix porcine genome array (platform GPL3533) were collected from GEO (http://www.ncbi.nlm.nih.gov/geo/) and ArrayExpress (http://www.ebi.ac.uk/arrayexpress/) databases. Datasets were filtered based on the following criteria: 1) availability of raw data (.cel files) and 2) a pathogen/pathogen component treatment challenge was part of the experimental design. All raw data (.cel files) were downloaded and the probe set expression levels were estimated using the robust multi-array average (RMA) method [74]. The quality of the raw data from each dataset was analyzed using the arrayQualityMetrics package in Bioconductor (http://www.bioconductor.org/) and scored on the basis of 5 metrics, namely maplot, spatial, boxplot, heatmap and rle. Any array failing on more than one metric was removed from the dataset. The collected data included four unpublished datasets from the pig research community (one from CKT and three from DAH/TAA; three datasets are now published: E-MTAB-505 [75]; GSE30956 [36]; and GSE27000 [34]). In total, 188 chips across 8 experiments fit this criterion, and were used as input for the cluster analysis (Additional file 5: Table S5). To find genes that responded similarly to an infection or stimulus across all groups, we used Robinson’s within-group correlation (Equation 1, [76]) to calculate pair-wise correlations for each probeset. These correlations emphasized similar responses to the known stimulus present in each dataset. A tgf matrix was created using a minimum correlation of 0.52, which empirically maximized cluster size while retaining functional annotation enrichment. The tgf file was then imported into BioLayout *Express*^3D^[38] and these correlations were used to create a co-expression graph.

Equation 1. Robinson’s within-groups correlation. The within-group correlation (*r*_*w*_) is a weighted average of the *j* within-group individual correlations between *X* and *Y*. Each within-group correlation was weighted by the size of the group for which it was calculated [76].

$$r_{w}=\sum_{j=1}^{k}r_{j}w_{j}=\sum_{j=1}^{k}\frac{\sum_{i=1}^{n_{j}}\left(X_{\mathrm{ij}}-{\bar{X}}_{j}\right)\left(Y_{\mathrm{ij}}-{\bar{Y}}_{j}\right)}{\sqrt{\sum_{i=1}^{n_{j}}{\left(X_{\mathrm{ij}}-{\bar{X}}_{j}\right)}^{2}}\sqrt{\sum_{i=1}^{n_{j}}{\left(Y_{\mathrm{ij}}-{\bar{Y}}_{j}\right)}^{2}}}\frac{n_{j}}{n}$$

Further analysis of the correlation graphs was then performed within BioLayout *Express*^3D^. In this context, nodes represented individual probesets (genes/transcripts) and the edges represented Pearson correlation coefficients between them. The network was clustered into groups of genes sharing similar profiles using the MCL algorithm with an empirical MCL inflation value (1.8) and a global graph for each network was created showing the MCL clusters. Several functions within BioLayout *Express*^3D^ were then used to explore and characterize the clusters created. In addition, cluster gene lists were exported and annotated using R scripts and GO-SLIM tools (at http://www.ebi.ac.uk) to find over-representation of GO terms and other functional annotations. Significance of enrichment of annotations was calculated using a modified Fisher’s exact test [77]. False discovery rate for this enrichment was also calculated and presented [78].

### Positive selection analysis

We focused on one cluster (number 4), built during the co-expression analyses above and shown to be enriched in immune response genes, to estimate the rate of evolutionary change for porcine immune genes. Within cluster 4 probesets, we were able to identify 295 unique genes with Ensembl IDs. From this set of 295 genes, 277 proteins annotated in Ensembl release 67 [79] were extracted. Some of these proteins result from alternative splicing; to maximize comparisons, we retained only the longest protein. Thus, a subset of 251 proteins was studied for evidence of positive selection, using the PhyleasProg phylogenetic analysis web server (http://phyleasprog.inra.fr/; [67]). Based on Ensembl release 67, PhyleasProg enables users to reconstruct phylogenetic trees, calculate positive selection with a visualization of these results on the protein sequence and on a 3D structure where possible, and explore genomic environment of query genes. Evolutionary analyses were carried out using 19 species (Chicken, Chimpanzee, Cow, Dog, Frog, Fugu, Horse, Human, Macaque, Medaka, Mouse, Opossum, Platypus, Pig, Rat, Stickleback, Tetraodon, Zebra finch and Zebrafish). Two runs of computations were done on the 251 proteins from Cluster 4, and evolution analyses were done through the same 19 species**.** We are fully aware that identification of actual positive selection events is a significant issue. Thus, to avoid overestimating the number of genes with an accelerated rate of evolution, we retained only the results from methods used with high stringency criteria. First, we used parameters “orthologs only” and “Fine computation” and second used parameters “orthologs only” and “Fast computation”. The Fine and Fast options correspond to two multiple sequence alignments methods, Prank (http://www.ebi.ac.uk/goldman-srv/prank/) and MUSCLE [80], respectively. Positive selection results were computed with site models (Model 1a vs. 2a, Model 7 vs. 8 and Model 8a vs. 8) and branch-site models of PAML [81]. Branch-site models were designed to detect signals of local episodic positive selection in order to determine whether different species underwent selective pressure. Bayes Empirical Bayes (BEB) method implemented in PAML was used to estimate posterior probabilities of selection on each codon, and probabilities > 0.95 were considered significant. After the Prank alignment, multiple sequence alignments were refined by GBLOCKS [82], improved by a home-made Perl program. All positive selection results found specific to pig were checked manually. In the context of multiple testing, we calculated q value measures as an extension of the false discovery rate, using the q value package of R. The q value attached to each individual branch described the expected proportion of false positives among all branches equal to or more extreme than the observed one. Therefore, the thresholding of the estimated q values at alpha level =5% produced a list of significant branches so that the expected proportion of false positives was alpha. For the modeling of the 3D structure, a BLAST [83] was performed to find an approaching structure in PDB database [84] in order to use it as a template to calculate a model with MODELLER [85]. If a PDB sequence matched correctly with submitted protein, evolutionary results were directly visualized onto its modeled structure. The amino acids found under positive selection were mapped on potentially functional domains for the proteins CASP8, GBP1, HEXB and PPP2R5C. The sequence of each protein was submitted to InterProScan (http://www.ebi.ac.uk/Tools/pfa/iprscan/). This InterPro (http://www.ebi.ac.uk/interpro/) resource provided functional analysis of protein sequences by classifying them into families and predicting the presence of domains and important sites.

## Availability of supporting data

The data sets supporting the results of this article are available in the GEO or ArrayExpress databases under the dataset identifiers provided in the text. Gene structural annotations and sequences are available in Ensembl porcine build 10.2.

## Competing interests

The authors declare they have no competing interests.

## Authors’ contributions

CKT, CRG and HDD coordinated the Immune Response Annotation Group. The following authors provided manual annotation: JEL, HU, KMM, YS, JZ, DC-S, TH, MH, SZ, A. Anselmo, HS, CC, B. Badaoui, DB, TM, RPYC, MB, RK, JCS, RK, DT, A. Archibald, B. Bed’Hom, D. Beraldi, EG, JKL, JMR, CRG, and CKT. The following authors provided support for annotators: HDD, HU, SZ, FB, SB, EG, DAH, JKL, MPM, JLH, CRG, and CKT. JGRG, ZH, MA, EF, and JMR provided informatics support for manual annotation. JEL and JLH coordinated the Sanger annotation and QC teams. The following authors provided quality control of manual annotation: CA, MK, DL, C. Snow, JEL, C. Steward, MT, LW, JKL. JMR provided training with Otterlace to annotators. HDD performed the sequence comparisons of genes/gene families across species, and determined and/or collated the gene duplication and missing genes analyses. GP performed the phylogenetic analyses. THH and CKT performed the Biolayout Express^3D^ analyses with training from TCF, RK and DAH. CKT, DAH, and TAA provided datasets for Biolayout Express^3D^ analyses. HDD, CRG, CKT, GP, JEL, JCS, and MPM wrote the paper. All authors read and approved the final manuscript.

## Supplementary Material

Additional file 1: Table S1 Pig-human-mouse-bovine gene family comparisons. Click here for file

Additional file 2: Table S2 Known gene sequences that are missing from Build 10.2. Click here for file

Additional file 3: Table S3 True duplicated genes in Build 10.2. Click here for file

Additional file 4: Table S4 Artifactually duplicated genes in Build 10.2. Click here for file

Additional file 5: Table S5 List of datasets used in immune response clustering analysis. Click here for file

Additional file 6: Table S6 Gene Ontology annotation of Biolayout Express^3D^ clusters from immune response network shown in Figure 2. Click here for file

Additional file 7: Table S7 Summary of annotations of selected clusters from immune response clustering analysis. Click here for file

Additional file 8: Figure S1 Expression pattern of MCL cluster 17 shows gene activation after immune stimulation/infection in specific experimental datasets. In blue is shown the average expression of the 81 probesets in cluster 17. Details on each dataset are shown below the graph; some example patterns are highlighted in red. See abbreviations in legend to Figure 3. Click here for file

Additional file 9: Figure S2 Expression pattern of MCL cluster 26 shows gene expression common to Lymph Node and blood datasets without a strong pattern related to immune stimulation. In brown is shown the average expression of the 48 probesets in cluster 26. Details on each dataset are shown below the graph. See abbreviations in legend to Figure 3. Click here for file

Additional file 10: Figure S3 Expression pattern of MCL cluster 48 shows gene activation after immune stimulation/infection in many experimental datasets. In orange is shown the average expression of the 24 probesets in cluster 26. Details on each dataset are shown below the graph; some example patterns are highlighted in red. See abbreviations in legend to Figure 3. Click here for file

Additional file 11: Table S8. Results of positive selection computation on the 242 pig proteins from MCL cluster 4. Click here for file

Additional file 12: Figure S4. Results of positive selection calculation are visualized on primary sequence of (A) PPP2R5C, (B) GBP1, (C) HEXB and (D) CASP8 pig proteins. Amino acids in green font are under purifying selection. Amino acids in orange and red font are under positive selection with posterior probabilities greater than 95% or 99%, respectively. Amino acids in white font target those for which no information is available (no calculation was performed by PAML due to at least one gap in the multiple sequence alignment at this position). Amino acids are in grey font where results are not significant enough to infer either purifying or positive selection. Protein domains, as predicted by InterPro resources (see Methods) are represented by colored bars under amino acid sequences. Click here for file
