# Supplementary material for: Structural and functional annotation of the porcine immunome
Source: BMC Genomics. 2013 May 15;14:332. doi: 10.1186/1471-2164-14-332 (PMC3658956; doi:10.1186/1471-2164-14-332)
Supplement: Additional file 3: Table S3 — True duplicated genes in Build 10.2. [file 1471-2164-14-332-S3.doc]

**Supplementary Table 3. True duplicated genes in Build 10.2**

**Immune Related**

|  |  |  |  |  |  |
| --- | --- | --- | --- | --- | --- |
| **Gene** | **NCBI Loci** | **Chr** | **Build 10.2 Gene ID** | **Build 10.2 Gene Coordinates** | **Comments** |
| CD36 | 733702 | 9 | ENSSSCG00000015405 | NC_010451.3 :110040077-110102645 | Sequences in Unigene Ssc.35355 do not align to an identified gene in Ensembl build 9.2 but do align in build 10.2f. |
| CD36L1 | 100511343 | 9 | NA | NC_010451.3 :109954298-110015639 | Duplicated in cow. |
|  |  |  |  |  |  |
| CRP | 396842 | 4 | ENSSSCG00000006403 | NC_010446.4 :98764964-98772727 | Gene is not in build 9.2 but is in build 10.2f. |
| CRPL1 | 100620468 | 4 | ENSSSCG00000021186 | NC_010446.4 :98745242-98757413 | Porcine-specific gene. |
|  |  |  |  |  |  |
| IL1B | 397122 | 3 | ENSSSCG00000008088 | NC_010445.3 :45319296-45326287 |  |
| IL1BL1 | 396565 | 3 | ENSSSCT00000008860 | NC_010445.3 :45179378-45185849 | Porcine-specific gene. |
|  |  |  |  |  |  |
| CD68 | 100522571 | 12 | ENSSSCG00000017956 | NC_010454.3 :55296986-55299419 | Gene on porcine chromosome 12 is syntenic with human CD68 on chromosome 17. |
| CD68L1 | 100520753 | 8 | ENSSSCG00000008769 | NC_010450.3 :29672313-29674595 | Gene on porcine chromosome 8 is syntenic with human chromosome 4. |
|  |  |  |  |  |  |
| CD163 | 397031 | U | NA | NW_003539177.1 :5-471 | Sequences in Unigene Ssc.5053 partially align in Ensembl build 10.2. |
| CD163L1 | 100144477 | 5 | NA | NC_010447.4 :65984979-66021167 | CD163L1 is duplicated in cows, humans and mice. |
| CD163L2 | 100627089 | 5 | NA | NC_010447.4 :66030456-66085371 |  |
|  |  |  |  |  |  |
| IFIT1 | 100153038 | 14 | ENSSSCG00000010453 | NC_010456.4 :110223576-110235661 |  |
| IFIT1L1 | 100621926 | 1 | NA | NC_010443.4 :296319106-296331356 |  |
|  |  |  |  |  |  |
| ITLN2 | 100144619 | 4 | ENSSSCG00000028374 | NC_010446.4: 97610153-97621638 |  |
| ITLN2L | 100736733 | 4 | ENSSSCG00000025885 | NC_010446.4: 97403439-97430022 |  |
|  |  |  |  |  |  |
| DDX3X | 100515940 | X | ENSSSCG00000012252 | NC_010461.4: 41162035-41177759 |  |
| DDX3XL | 100624590 | Y | ENSSSCG00000026430 | NC_010462.2: 1115792-1132907 |  |
|  |  |  |  |  |  |
| GZMA | 100526762 | 16 | ENSSSCG00000016902 | NC_010458.3: 36385694-36393956 | Gene also duplicated in cow. |
| GZMAL | 100233183 |  |  | NC_010458.3: 36366028-36375618 |  |
|  |  |  |  |  |  |
| LY9 | 100156074 | 4 | ENSSSCG00000006375 | NC_010446.4: 97496457-97509189 |  |
| LY9L1 | 100525572 | 4 | ENSSSCG00000006376 | NC_010446.4: 101230146-101243685 | Gene erroneously annotated as SLAMF9 by NCBI. |
|  |  |  |  |  |  |
| IRGC | 100462753 | 6 | ENSSSCG00000003066 | NC_010448.3: 46399976-46403321 | Gene also duplicated in cow. |
| IRGCL | 100523557 | 6 | ENSSSCG00000003067 | NC_010448.3: 46403760-46409267 |  |
|  |  |  |  |  |  |
| GSTP1 | 100739163 | 2 | ENSSSCG00000012897 | NC_010444.3: 3618596-3623836 |  |
| GSTP1L1 | 100525731 | 2 | ENSSSCG00000012901 | NC_010444.3: 3724620-3731427 | Gene is a duplicate of GSTP1 and is present in cow and mouse but not human. |
|  |  |  |  |  |  |
| ATF4 | 100144302 | 5 | ENSSSCG00000012860 | NC_010447.4: 6042584-6044651 |  |
| ATF4L | Not Assigned | 2 | ENSSSCG00000012860 | Not Assigned |  |
|  |  |  |  |  |  |
|  |  |  |  |  |  |
| **Non-immune Related** | | | | | |
|  |  |  |  |  |  |
| RDH16 | 100511633 | 5 | ENSSSCG00000000414 | NC_010447.4 24133013-24139006 | Artiodactyl-and Perissodactyl-specfic duplication, there are 3 putative bovine and 3 equine genes that are similar to RDH16. |
| RDH16L1 | 100512656 | 5 | NA | NC_010447.4 24087398-24094529 | Sequences in Unigene Ssc.94004 or Ssc.55153 do not align to an identified gene in Ensembl build 9. |
|  |  |  |  |  |  |
| ABCC4 | 100152536, 100627251 | 11 | ENSSSCG00000009495 | NC_010453.4 70384867-70481706 |  |
| ABCC4L1 | 100518213 | 11 | ENSSSCG00000009496 | NC_010453.4 70779814-70852608 |  |
| ABCC4L2 | 100517850 | 11 | ENSSSCG00000009498 | NC_010453.4 70591445-70749846 |  |
| ABCC4L3 | 100518860 | 11 | ENSSSCG00000022549 | NC_010453.4 71059813-71227114 |  |
|  |  |  |  |  |  |
| CYP19A1 | 403331 |  |  | NW_003540886.1 |  |
| CYP19A2 | 403332 | 1 |  | NC_010443.4 133903926-133936093 |  |
| CYP19A3 | 403333 | 1 |  |  |  |
|  |  |  |  |  |  |
| FOLR1 | 397579 | 9 | ENSSSCG00000014810 | NC_010451.3 7515002-7521922 |  |
| FOLR1L1 | 396784 | 9 | ENSSSCG00000022236 | NC_010451.3 7507564-7511915 |  |
|  |  |  |  |  |  |
| EIF5 | Not Assigned | U |  | Not present. | Sequences in Unigene Ssc.54686 imperfectly align to ENSSSCG00000027421. |
| EIF5L | 100522498 | 6 | ENSSSCG00000027421 | NC_010448.3 137319490..137326609 | No EST or other evidence for transcription, gene is also artifactually duplicated. Predicted protein is 74% identical to EIF5. |
