# Supplementary material for: Structural and functional annotation of the porcine immunome
Source: BMC Genomics. 2013 May 15;14:332. doi: 10.1186/1471-2164-14-332 (PMC3658956; doi:10.1186/1471-2164-14-332)
Supplement: Additional file 8: Figure S1 — Expression pattern of MCL cluster 17 shows gene activation after immune stimulation/infection in specific experimental datasets. In blue is shown the average expression of the 81 probesets in cluster 17. Details on each dataset are shown below the graph; some example patterns are highlighted in red. See abbreviations in legend to Figure 3. [file 1471-2164-14-332-S8.pptx]

## Slide 1
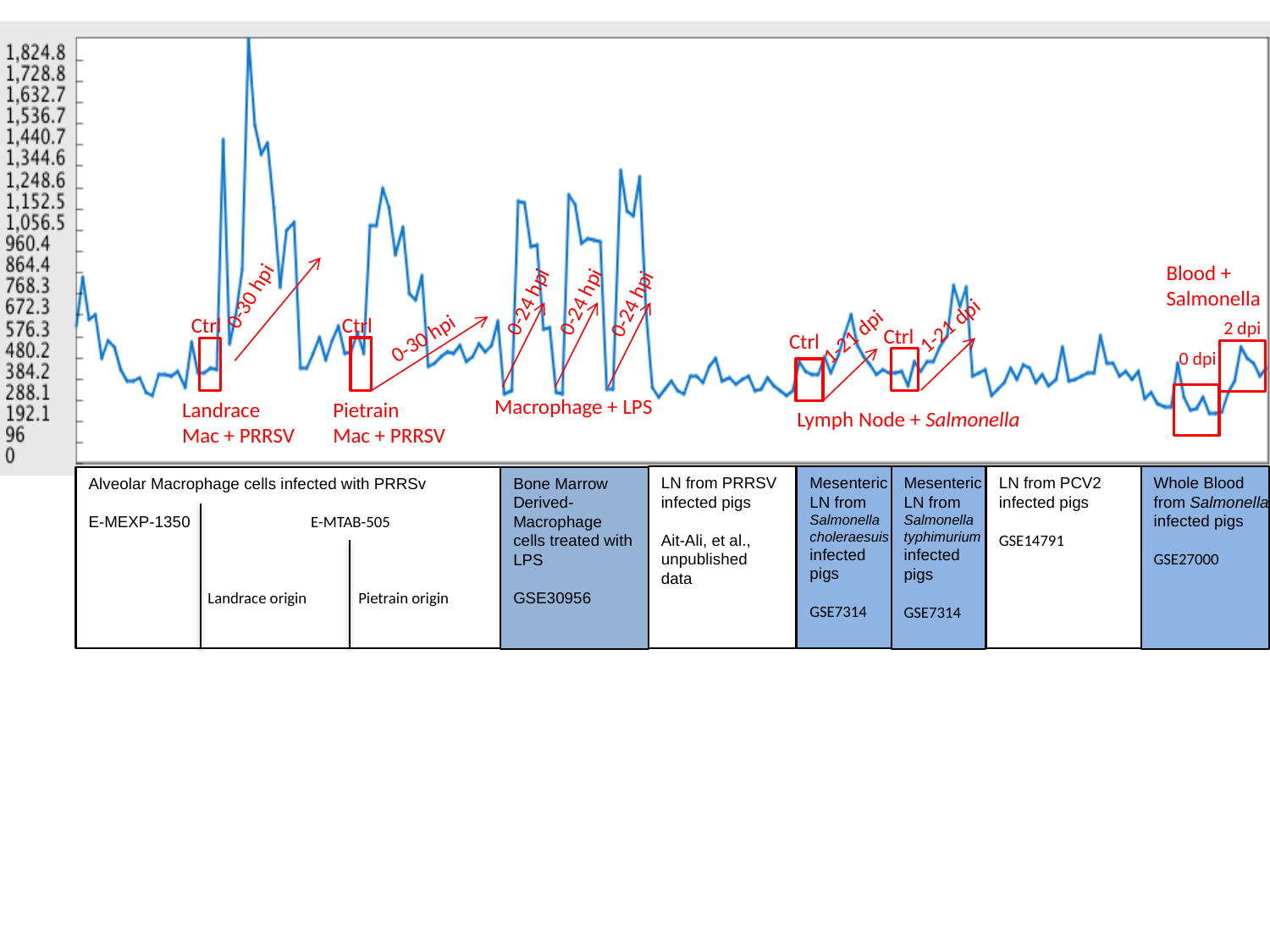

Blood + Salmonella
0-24 hpi
0-24 hpi
0-24 hpi
Macrophage + LPS
0-30 hpi
Ctrl
0-30 hpi
Pietrain
Mac + PRRSV
Ctrl
1-21 dpi
2 dpi
1-21 dpi
Ctrl
Ctrl
0 dpi
Landrace
Mac + PRRSV
Lymph Node + Salmonella
Whole Blood from Salmonella infected pigs
GSE27000
LN from PRRSV infected pigs
Ait-Ali, et al.,
unpublished
data
Mesenteric LN from Salmonella choleraesuis infected pigs
GSE7314
LN from PCV2 infected pigs
GSE14791
Mesenteric LN from Salmonella typhimurium infected pigs
GSE7314
Alveolar Macrophage cells infected with PRRSv
E-MEXP-1350 	E-MTAB-505
	Landrace origin	Pietrain origin
Bone Marrow Derived-Macrophage cells treated with LPS
GSE30956
