# Supplementary material for: Structural and functional annotation of the porcine immunome
Source: BMC Genomics. 2013 May 15;14:332. doi: 10.1186/1471-2164-14-332 (PMC3658956; doi:10.1186/1471-2164-14-332)
Supplement: Additional file 9: Figure S2 — Expression pattern of MCL cluster 26 shows gene expression common to Lymph Node and blood datasets without a strong pattern related to immune stimulation. In brown is shown the average expression of the 48 probesets in cluster 26. Details on each dataset are shown below the graph. See abbreviations in legend to Figure 3. [file 1471-2164-14-332-S9.pptx]

## Slide 1
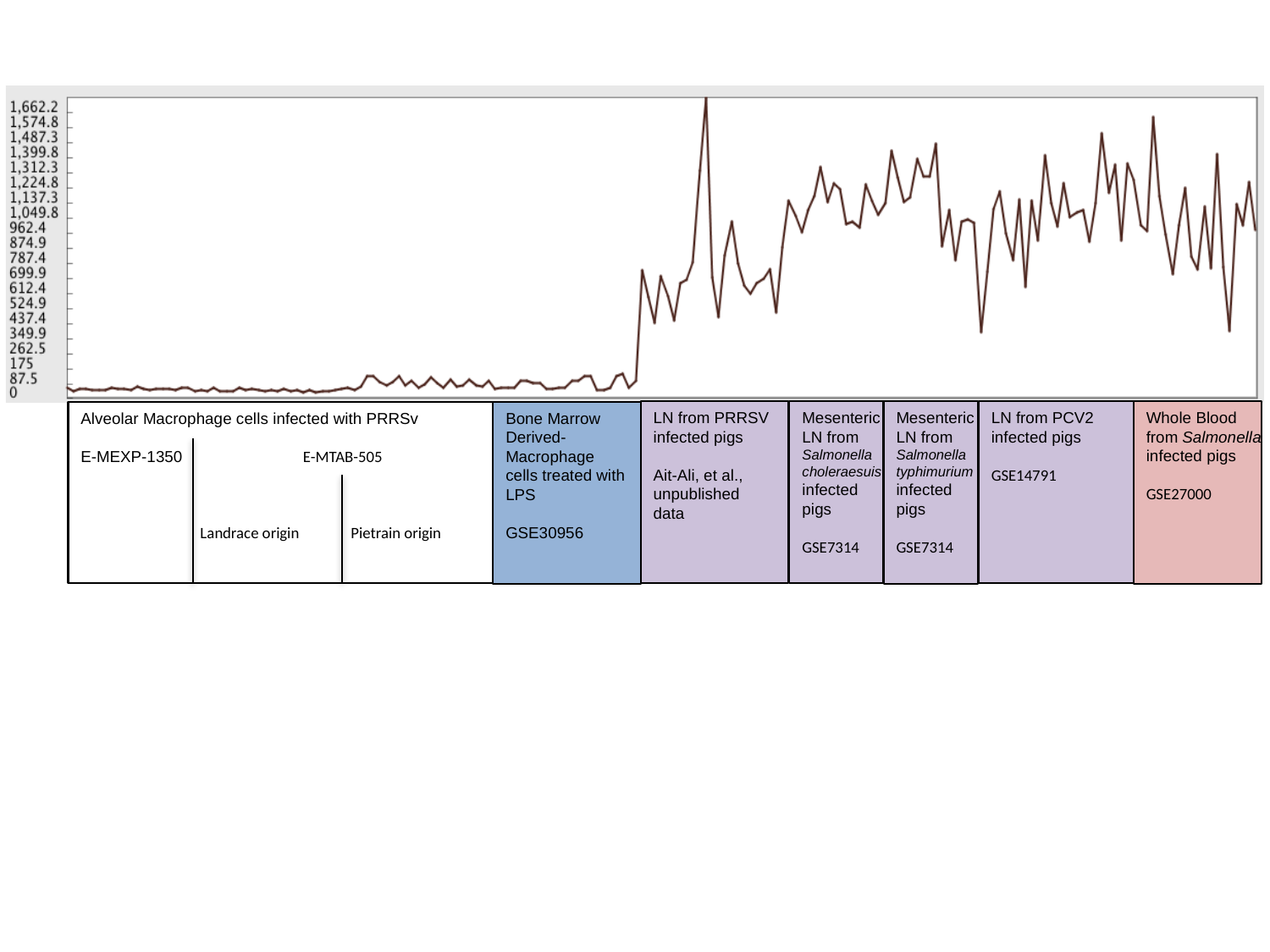

Whole Blood from Salmonella infected pigs
GSE27000
LN from PRRSV infected pigs
Ait-Ali, et al.,
unpublished
data
Mesenteric LN from Salmonella choleraesuis infected pigs
GSE7314
LN from PCV2 infected pigs
GSE14791
Mesenteric LN from Salmonella typhimurium infected pigs
GSE7314
Alveolar Macrophage cells infected with PRRSv
E-MEXP-1350 	E-MTAB-505
	Landrace origin	Pietrain origin
Bone Marrow Derived-Macrophage cells treated with LPS
GSE30956
