# Supplementary material for: Structural and functional annotation of the porcine immunome
Source: BMC Genomics. 2013 May 15;14:332. doi: 10.1186/1471-2164-14-332 (PMC3658956; doi:10.1186/1471-2164-14-332)
Supplement: Additional file 12: Figure S4. — Results of positive selection calculation are visualized on primary sequence of (A) PPP2R5C, (B) GBP1, (C) HEXB and (D) CASP8 pig proteins. Amino acids in green font are under purifying selection. Amino acids in orange and red font are under positive selection with posterior probabilities greater than 95% or 99%, respectively. Amino acids in white font target those for which no information is available (no calculation was performed by PAML due to at least one gap in the multiple sequence alignment at this position). Amino acids are in grey font where results are not significant enough to infer either purifying or positive selection. Protein domains, as predicted by InterPro resources (see Methods) are represented by colored bars under amino acid sequences. [file 1471-2164-14-332-S12.pptx]

## Slide 1
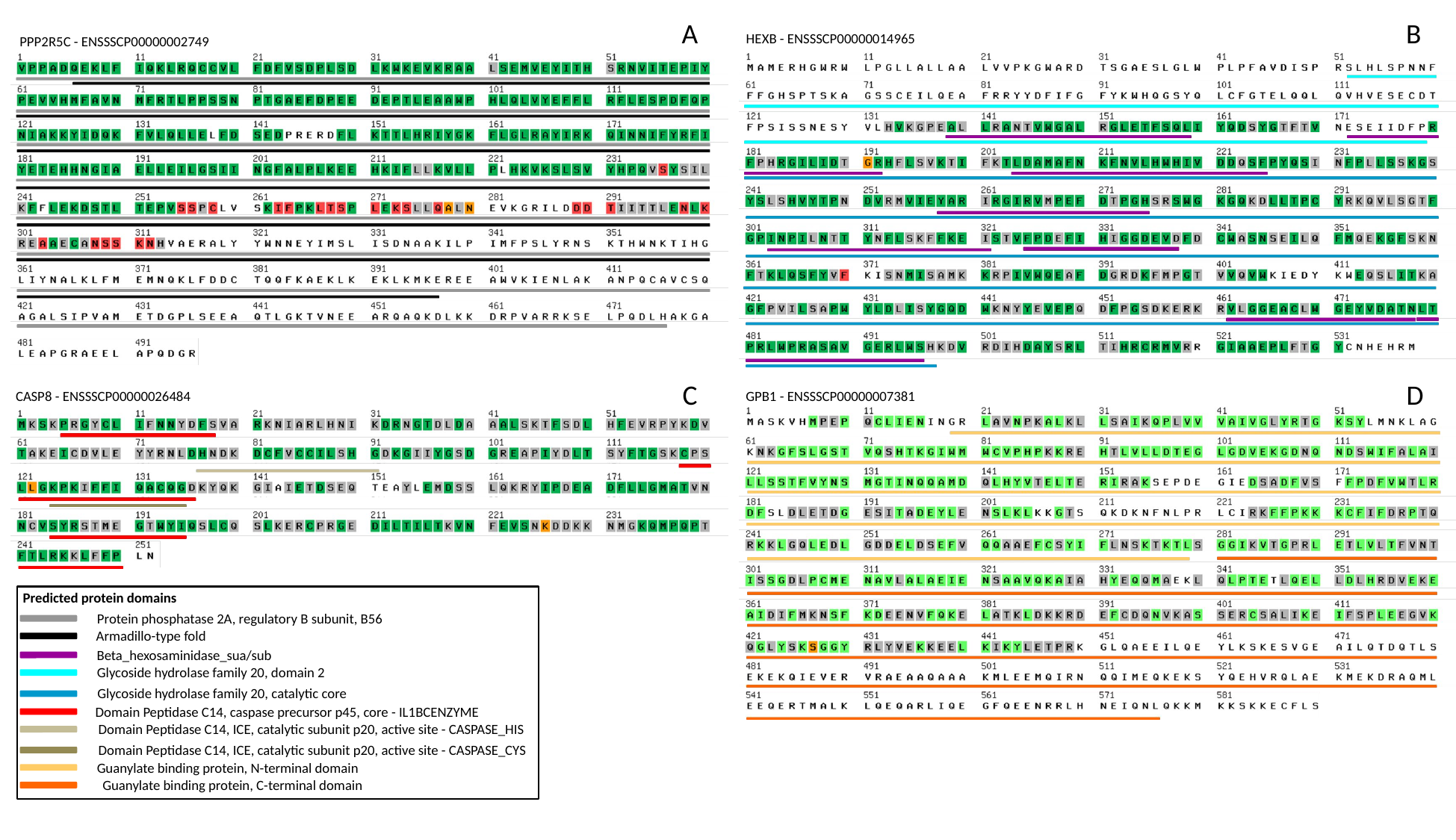

A
B
HEXB - ENSSSCP00000014965
PPP2R5C - ENSSSCP00000002749
C
D
CASP8 - ENSSSCP00000026484
GPB1 - ENSSSCP00000007381
Predicted protein domains
Protein phosphatase 2A, regulatory B subunit, B56
Armadillo-type fold
Beta_hexosaminidase_sua/sub
Glycoside hydrolase family 20, domain 2
Glycoside hydrolase family 20, catalytic core
Domain Peptidase C14, caspase precursor p45, core - IL1BCENZYME
Domain Peptidase C14, ICE, catalytic subunit p20, active site - CASPASE_HIS
Domain Peptidase C14, ICE, catalytic subunit p20, active site - CASPASE_CYS
Guanylate binding protein, N-terminal domain
Guanylate binding protein, C-terminal domain
